# Unexpected implantation of cardiac resynchronization therapy: Its characteristics and prognosis

**DOI:** 10.1016/j.hroo.2024.09.004

**Published:** 2024-09-16

**Authors:** Naoya Kataoka, Teruhiko Imamura, Koichiro Kinugawa

**Affiliations:** Second Department of Internal Medicine, University of Toyama, Toyama, Japan

**Keywords:** Heart failure, Cardiac resynchronization therapy, Unexpected implantation, Electrophysiologically unexpected, Hemodynamically unexpected

**Key Findings**

▪ The clinical safety and implications of unexpected cardiac resynchronization therapy (CRT) implantation remain inadequately understood.
▪ The safety of unexpected CRT implantation in electrophysiologically or hemodynamically unstable conditions was comparable to that of elective procedures.
▪ Given the high incidence of adverse events in hemodynamically unstable patients, physicians should carefully evaluate the optimal timing for CRT implantation throughout the course of heart failure management.

The majority of previous discussions regarding cardiac resynchronization therapy (CRT) have focused on parameters in elective situations, with limited information on implantation timing and its clinical impacts.^1^ This study investigated real-world data on the timing of CRT implantation—whether unexpected or elective—and its associated clinical characteristics, by comparing the safety and efficacy of unexpected versus elective implantations.

In this retrospective study, consecutive patients who underwent CRT implantation at our institute from March 2010 to March 2024 were analyzed. Patients were classified into 3 groups based on the reasons for CRT implantation: (1) the electrophysiologically unexpected group comprised patients with sudden atrioventricular conduction disorders and systolic heart failure; (2) the hemodynamically unexpected group comprised patients requiring CRT implantation during hospitalization due to worsening heart failure or cardiogenic shock; and (3) the elective group comprised patients undergoing planned outpatient implantation. Three components were compared among the groups: (1) the safety of unplanned implantations, indicated by complication rates and procedure duration; (2) the echocardiographic response to CRT 6 months postimplantation, categorized as worsened, unchanged, or improved per a previous study; and (3) the clinical events following CRT, including all-cause death, durable left ventricular assist device implantation, and hospitalization for worsening heart failure.^2^

A total of 131 subjects were included in the analysis, categorized into 3 groups: 22 (17%) in the electrophysiologically unexpected group, 37 (28%) in the hemodynamically unexpected group, and 72 (55%) in the elective group. Their baseline characteristics, detailed in Table 1, showed significant differences among the groups in several variables, including New York Heart Association functional class, administration of loop diuretics and inotropes, renal function, and plasma B-type natriuretic peptide levels.

**Table 1:** Baseline characteristics

Variable	Overall	Electrophysiologically unexpected group	Hemodynamically unexpected group	Elective group	*P* Value
Age at device implantation, y	74 (65–81)	70 (63–83)	73 (65–82)	75 (65–81)	.93
Female	49 (37)	8 (36)	15 (41)	26 (36)	.90
Body mass index, kg/m^2^	20.5 (18.2–23.8)	22.0 (18.2–23.8)	19.8 (17.9–22.0)	20.9 (18.7–24.2)	.23
Etiology					
Ischemic heart disease	22 (17)	4 (18)	8 (22)	10 (14)	.58
Dilated cardiomyopathy	42 (32)	3 (14)	15 (41)	24 (33)	.095
Sarcoidosis	17 (13)	3 (14)	3 (8)	11 (15)	.57
Valvular heart disease	14 (11)	5 (23)	2 (5)	7 (10)	.11
With implantable cardioverter-defibrillator	92 (70)	17 (77)	27 (73)	48 (67)	.58
New York Heart Association functional class					<.001
II	66 (50)	11 (50)	8 (22)	47 (65)	
III	50 (38)	8 (36)	20 (54)	22 (31)	
IV	15 (11)	3 (14)	9 (24)	3 (4)	
*Medications*					
ACE inhibitor, ARB, or ARNI	117 (89)	19 (86)	31 (84)	67 (93)	.30
β-blockers	105 (80)	16 (73)	31 (84)	58 (81)	.58
Mineralocorticoid receptor antagonists	78 (60)	14 (64)	22 (59)	42 (58)	.91
Sodium-glucose cotransporter 2 inhibitor	35 (27)	8 (36)	9 (24)	18 (25)	.53
Loop diuretics	96 (73)	10 (45)	32 (86)	54 (75)	.002
Inotropes	26 (20)	5 (23)	14 (38)	7 (10)	.002
QRS morphologies					.34
Left bundle branch block	58 (44)	7 (32)	19 (51)	32 (44)	.34
Right bundle branch block	11 (8)	0	5 (14)	6 (8)	.19
Interventricular conduction delay	20 (15)	6 (27)	8 (22)	6 (8)	.043
Right ventricular pacing form	42 (32)	9 (41)	5 (14)	28 (39)	.020
Echocardiographic parameters					
Left ventricular end-diastolic diameter, mm	59 (54–66)	54 (48–62)	63 (56–72)	59 (54–66)	.001
Left ventricular end-systolic diameter, mm	49 (43–58)	42 (37–49)	53 (47–66)	49 (44–58)	<.001
Left ventricular ejection fraction, %	31 (24–36)	35 (30–43)	25 (20–33)	32 (25–37)	<.001
Laboratory data					
Serum creatinine, mg/dL	1.1 (0.8–1.5)	1.1 (0.9–1.7)	1.4 (0.9–2.3)	1.0 (0.8–1.3)	.003
Plasma B-type natriuretic peptide, pg/mL	259 (129–649)	322 (113–607)	590 (198–1004)	185 (110–421)	.001

Values are median (interquartile range) or n (%).

ACE = angiotensin-converting enzyme; ARB = angiotensin receptor blocker; ARNI = angiotensin receptor neprilysin inhibitor.

The median operation durations were 120 (interquartile range [IQR] 93–148) minutes for the electrophysiologically unexpected group, 130 (IQR 103–168) minutes for the hemodynamically unexpected group, and 135 (IQR 110–160) minutes for the elective group, with no significant differences (*P* = .26). Complications occurred in 5 (3.8%) patients: 1 required a blood transfusion due to bleeding, 1 had a coronary vein perforation that required no treatment, and 3 developed pocket hematomas that did not need invasive treatment. No complications occurred in the electrophysiologically unexpected group, 2 occurred in the hemodynamically unexpected group, and 3 occurred in the elective group (*P* = .56).

Regarding echocardiographic responses to CRT, 11% of the electrophysiologically unexpected group showed improvement, while 44% remained unchanged; in the hemodynamically unexpected group, 43% showed improvement and 33% remained unchanged; and in the elective group, 65% showed improvement and 25% remained unchanged (*P* = .021) (Figure 1).

**Figure 1:**
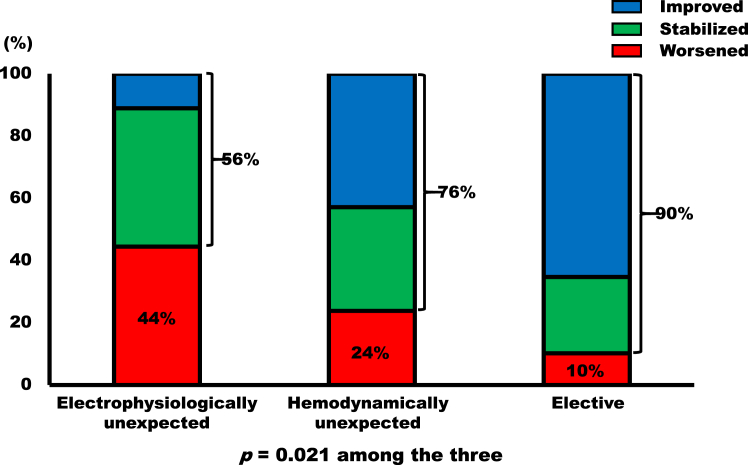
The reduction of left ventricular end-systolic volume index at follow-up.

During a median follow-up of 2.0 years (interquartile range: 1.0–3.9 years) after CRT implantation, 33 subjects experienced composite adverse events. At 1 year post-implantation, the electrophysiologically unexpected group had outcomes comparable to the hemodynamically unexpected group but worse than the elective group. However, after 1 year, no adverse events occurred in the electrophysiologically unexpected group, and their composite event-free rates became comparable to those in the elective group. In contrast, the hemodynamically unexpected group experienced progressively worse outcomes over time (*P* < .001) (Figure 2).

**Figure 2:**
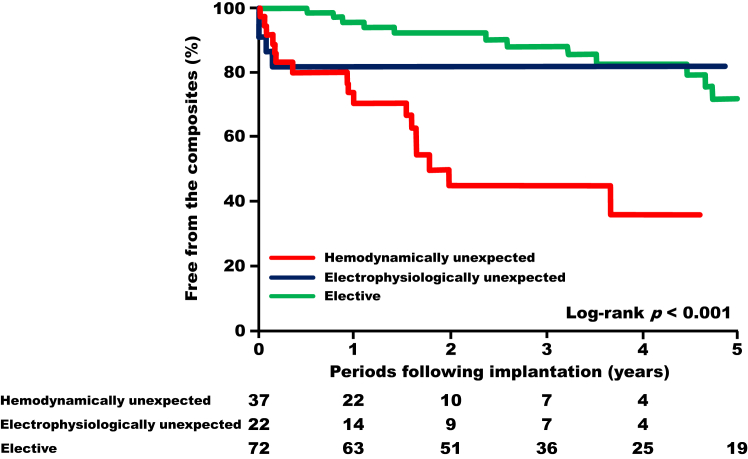
Kaplan-Meier curves of the composite adverse events.

The study's findings indicate that procedural safety was statistically comparable among the 3 groups. The electrophysiologically unexpected group exhibited less left ventricular reverse remodeling but did not have significantly worse clinical outcomes compared with the elective group. Conversely, the hemodynamically unexpected group showed over 70% improvement or stabilization in left ventricular end-systolic volume index but had poorer outcomes than the elective group.

Electrophysiologically unexpected implantation demonstrated no statistical inferiority to the elective group regarding procedural safety and adverse events, supporting the recommendation for unexpected CRT implantation over pacemaker placement. However, the hemodynamically unexpected group had worse outcomes compared with the elective group, indicating that patients hospitalized for worsening heart failure or cardiogenic shock may face poorer outcomes following CRT implantation. This highlights the issue of CRT underuse, as untreated populations often experience adverse outcomes, including higher all-cause mortality and heart failure-related hospitalizations.^3^ The hemodynamically unexpected group may represent those affected by this underuse.

Contrary to the response to CRT predicted by the survival curve, as many as 44% of cases in the electrophysiologically unexpected group were classified as having worsened (Figure 1). This finding may be attributed to the lower incidence of dilated cardiomyopathy and the higher incidence of sarcoidosis and valvular heart disease as underlying conditions, although there was no statistical significance (Table 1).

In conclusion, the safety of unexpected CRT implantation was comparable to that in elective subjects. Due to favorable outcomes observed in the electrophysiologically unexpected group, CRT implantation should not be delayed in favor of a pacemaker. Conversely, frequent adverse events were noted in the hemodynamically unexpected groups. Therefore, physicians should consistently consider appropriate timing for CRT implantation throughout heart failure therapies.

## Disclosures

The authors have no conflicts to disclose.
